# Supplementary material for: Vsb1, Ypq1, and Ypq2 control dynamic cationic amino acid storage in the yeast vacuole
Source: Life Sci Alliance. 2026 May 11;9(7):e202503520. doi: 10.26508/lsa.202503520 (PMC13160679; doi:10.26508/lsa.202503520)
Supplement: Supplementary file 1 [file LSA-2025-03520_TableS1.docx]

The doubling time of the yeast was measured in the w-t, *vsb1*Δ, *ypq1*Δ, *ypq1-2-3*Δ mutant strains complemented with the pFL38 plasmid, vsb1Δ mutant strain complemented with pFV438 plasmid and *ypq1*Δ mutant strain complemented with pFV445 plasmid. Data is reported as mean values with standard deviations (n = 3 biological replicates).

| **Strain** | **Genotype** | **G (hours)** |
| --- | --- | --- |
| 23344C pFL38 | *ura3* Ycp (URA3) | 2.17±0.02 |
| COM090 pFL38 | *ura3vsb1*Δ Ycp (URA3) | 2.09±0.01 |
| EL029 pFL38 | *ura3ypq1*Δ Ycp (URA3) | 2.21±0.03 |
| LL180 pFL38 | *ura3ypq1*Δ*ypq2*Δ*ypq3*Δ Ycp (URA3) | 2.11±0.02 |
| COM090 pFV438 | *ura3vsb1*Δ Ycp-TDH3p-VSB1 (URA3) | 2.09±0.03 |
| EL029 pFV445 | *ura3ypq1*Δ Ycp-TDH3p-YPQ1 (URA3) | 2.07±0.03 |
