# Supplementary material for: Vsb1, Ypq1, and Ypq2 control dynamic cationic amino acid storage in the yeast vacuole
Source: Life Sci Alliance. 2026 May 11;9(7):e202503520. doi: 10.26508/lsa.202503520 (PMC13160679; doi:10.26508/lsa.202503520)
Supplement: Supplementary file 3 [file LSA-2025-03520_TableS2.docx]

Strains used in this study

| **Strain** | **Genotype** | **Reference** |
| --- | --- | --- |
| Σ1278b derived strains | | |
| 23344c | *ura3* | Laboratory collection |
| FV1624 | *ura3 vph1Δ::KANMX6* | This study |
| LL162 | *ura3 vba1Δ::loxP vba2Δ::HPHMX4 vba3Δ::KANMX6* | (Cools et al., 2020) |
| COM090 | *ura3 vsb1Δ::HPHMX4* | (Jézégou et al., 2012) |
| EL029 | *ura3 ypq1Δ::KANMX6* | (Jézégou et al., 2012) |
| EL031 | *ura3 ypq2Δ::loxP* | (Jézégou et al., 2012) |
| EL043 | *ura3 ypq3Δ::KANMX6* | (Jézégou et al., 2012) |
| LL180 | *ura3 ypq1Δ::KANMX6 ypq2Δ::loxP ypq3Δ::HPHMX4* | (Jézégou et al., 2012) |
| FV1505 | *ura3 vsb1Δ::HPHMX4 ypq1Δ::NATMX6* | This study |
| 27038a | *ura3 npi1* | (Hein et al., 1995) |
| FV1402 | *ura3 npi1 vsb1Δ::KANMX6* | This study |
| COM148 | *ura3 lys2Δ::NATMX6* | This study |
| COM146 | *ura3 lys2Δ::NATMX6 ypq1Δ::KANMX6* | This study |
| FV1509 | *ura3 lys2Δ::NATMX6 ypq1Δ::KANMX6 ypq2Δ ypq3Δ::HPHMX4* | This study |
| FV1456 | *ura3 lys2Δ::KANMX6 vsb1Δ::HPHMX4* | This study |
| COM133 | *ura3 YPQ1-GFP-KANMX6* | This study |
| COM154 | *ura3 lys2Δ YPQ1-GFP-KANMX6* | This study |
| FV1626 | *ura3 npi1 vph1Δ::KANMX6* | This study |
| FV1662 | *ura3 npi1 vph1Δ::NATMX6 ypq1Δ::KANMX6* | This study |
| CG010 | *ura3 gap1Δ::loxP can1Δ::KANMX6 lyp1Δ::HPHMX4* | (Gournas et al., 2017) |
| BY4742 derived strains | | |
| FV1633 | *his3Δ1 leu2Δ0 lys2Δ0 ura3Δ0 arg4Δ::KANMX6* | This study |
| FV1636 | *his3Δ1 leu2Δ0 lys2Δ0 ura3Δ0 ypq1Δ::NATMX6 arg4Δ::KANMX6* | This study |
| FV1638 | *his3Δ1 leu2Δ0 lys2Δ0 ura3Δ0 ypq2Δ::HPHMX4 arg4Δ::KANMX6* | This study |
| FV1639 | *his3Δ1 leu2Δ0 lys2Δ0 ura3Δ0 ypq1Δ::NATMX6 ypq2Δ::HPHMX4 arg4Δ::KANMX6* | This study |
