# Supplementary material for: Vsb1, Ypq1, and Ypq2 control dynamic cationic amino acid storage in the yeast vacuole
Source: Life Sci Alliance. 2026 May 11;9(7):e202503520. doi: 10.26508/lsa.202503520 (PMC13160679; doi:10.26508/lsa.202503520)
Supplement: Supplementary file 4 [file LSA-2025-03520_TableS3.docx]

D5 and D3 oligonucleotides were used to amplify the cassette for the deletion of the corresponding gene.

5GFP and 3GFP oligonucleotides were used to amplify the cassettes for the insertion of GFP to the c-terminus of the ORF of the corresponding gene.

| **Oligonucleotide** | **Sequence** |
| --- | --- |
| D5 VPH1 | CAAAAAAAAAAAAACATTTAAAGGTTACACAAGGAAAATAcagctgaagcttcgtacgctgc |
| D3 VPH1 | AGTACTTAAATGTTTCGCTTTTTTTAAAAGTCCTCAAAGTgcggccgcataggccactag |
| D5 YPQ1 | TACAAAAAAAAAGCTACCACATCGCTCGACGACCTCAATTgctgaagcttcgtacgctgc |
| D3 YPQ1 | GAGGTCAAAAATATGTTAAATAAATATTAGATAGAACATGataggccactagtggatctg |
| D5 VSB1 | AGCAAGACAGTTTAAAATCTCAAAAATTTAATTTAGAAATgctgaagcttcgtacgctgc |
| D3 VSB1 | CCGGCAATAAAAGAAATTAAATAAATATACGTGTACGCTCgctgaagcttcgtacgctgc |
| D5 LYS2 | AACTGCTAATTATAGAGAGATATCACAGAGTTACTCACTAgcggccgccagctgaagctt |
| D3 LYS2 | TAATTATTGTACATGGACATATCATACGTAATGCTCAACCgcggccgcataggccactag |
| 5GFP YPQ1 | GTTTTTCATTTACAAAAGAAATAAAAAATTTATACTGAATggtgacggtgctggttta |
| 3GFP YPQ1 | GAGGTCAAAAATATGTTAAATAAATATTAGATAGAACATGtcgatgaattcgagctcg |
| D5 ARG4 | GCTCAAAAGCAGGTAACTATATAACAAGACTAAGGCAAACgctgaagcttcgtacgctgc |
| D3 ARG4 | CCAGACCTGATGAAATTCTTGCGCATAACGTCGCCATCTGataggccactagtggatctg |
| D5-YPQ2 | TCGGTGCTAAAAATTATAAAGTGAAAATAAGAATCTTTTCgctgaagcttcgtacgctgc |
| D3-YPQ2 | GCATTGTTTAAATATAAATGGTAGTACTCCGTAGGGAAAAgctgaagcttcgtacgctgc |
| 5D-YPQ3 | AGACGTAATAGCCTCAAGCGAGCATCCCTAAATTTCTGCCgctgaagcttcgtacgctgc |
| 3D-YPQ3 | GTTTGAGTTTTAAGTGGAAAGATTAATAGAAGCCAACTAGgctgaagcttcgtacgctgc |
