# Supplementary material for: Vsb1, Ypq1, and Ypq2 control dynamic cationic amino acid storage in the yeast vacuole
Source: Life Sci Alliance. 2026 May 11;9(7):e202503520. doi: 10.26508/lsa.202503520 (PMC13160679; doi:10.26508/lsa.202503520)
Supplement: Supplementary file 5 [file LSA-2025-03520_TableS4.docx]

Plasmids used in this study

| **Plasmid** | **Description** | **Reference** |
| --- | --- | --- |
| pFL38 | YCp (URA3) | (Bonneaud et al., 1991) |
| pFV438 | YCp-TDH3p-VSB1 (URA3) | This study |
| pFV445 | YCp-TDH3p-YPQ1 (URA3) | This study |
| pNAM001 | YCp-LYP1p-LYP1-GFP (URA3) | (Ghaddar et al., 2014) |
| pFV409 | YCp-VSB1p-VSB1 (URA3) | This study |
| pFV510 | YCp-VSB1p-VSB1^Δ1-171^ (URA3) | This study |
| pFV518 | YCp-VSB1p-VSB1^Δ639-1036^ (URA3) | This study |
| pFV511 | YCp-VSB1p-VSB1^Δ773-1036^ (URA3) | This study |
| pFV512 | YCp-VSB1p-VSB1^Δ849-1036^ (URA3) | This study |
| pFV452 | YCp-VSB1p-VSB1^D223A^ (URA3) | This study |
| pFV481 | YCp-VSB1p-VSB1^E275A^ (URA3) | This study |
| pFV526 | YCp-VSB1p-VSB1^E278A^ (URA3) | This study |
| pFV527 | YCp-VSB1p-VSB1^Y227A^ (URA3) | This study |
| pFV419 | YCp-VSB1p-VSB1-GFP (URA3) | This study |
| pFV514 | YCp-VSB1p-VSB1^Δ1-171^-GFP (URA3) | This study |
| pFV519 | YCp-VSB1p-VSB1^Δ639-1036^-GFP (URA3) | This study |
| pFV515 | YCp-VSB1p-VSB1^Δ773-1036^-GFP (URA3) | This study |
| pFV516 | YCp-VSB1p-VSB1^Δ849-1036^-GFP (URA3) | This study |
| pFV471 | YCp-VSB1p-VSB1^D223A^-GFP (URA3) | This study |
| pFV484 | YCp-VSB1p-VSB1^E275A^-GFP (URA3) | This study |
| pFV520 | YCp-VSB1p-VSB1^E278A^-GFP (URA3) | This study |
| pFV531 | YCp-VSB1p-VSB1^Y227A^-GFP (URA3) | This study |
