# Supplementary material for: Vsb1, Ypq1, and Ypq2 control dynamic cationic amino acid storage in the yeast vacuole
Source: Life Sci Alliance. 2026 May 11;9(7):e202503520. doi: 10.26508/lsa.202503520 (PMC13160679; doi:10.26508/lsa.202503520)
Supplement: Supplementary file 6 [file LSA-2025-03520_TableS5.docx]

Oligonucleotides used in this study for quantitative RT-PCR

| **Oligonucleotide** | **Sequence** | **Reference** |
| --- | --- | --- |
| LYS9-O1 | CTTACATCTCACCTGCCT | This study |
| LYS9-O2 | GTGGTCGATACCTGGATC | This study |
| TBP1-O1 | TATAACCCCAAGCGTTTTGC | (Georis et al., 2009) |
| TBP1-O2 | GCCAGCTTTGAGTCATCCTC | (Georis et al., 2009) |
